# Factitious lymphoedema as a psychiatric condition mimicking reflex sympathetic dystrophy: a case report

**DOI:** 10.1186/1752-1947-2-216

**Published:** 2008-06-24

**Authors:** Nnamdi Nwaejike, HAP Archbold, Darrin S Wilson

**Affiliations:** 1 Department of Vascular and Endovascular Surgery, Barts and The London NHS Trust, The Royal London Hospital, London, E1 1BB, UK; 2 Department of Fractures and Orthopaedics, The Royal Victoria Hospital, Grosvenor Road, Belfast, BT12 BA, UK

## Abstract

**Introduction:**

Reflex sympathetic dystrophy can result in severe disability with only one in five patients able to fully resume prior activities. Therefore, it is important to diagnose this condition early and begin appropriate treatment. Factitious lymphoedema can mimic reflex sympathetic dystrophy and is caused by self-inflicted tourniquets, blows to the arm or repeated skin irritation. Patients with factitious lymphoedema have an underlying psychiatric disorder but usually present to emergency or orthopaedics departments. Factitious lymphoedema can then be misdiagnosed as reflex sympathetic dystrophy. The treatment for factitious lymphoedema is dealing with the underlying psychiatric condition.

**Case presentation:**

We share our experience of treating a 33-year-old man, who presented with factitious lymphoedema, initially diagnosed as reflex sympathetic dystrophy.

**Conclusion:**

Awareness of this very similar differential diagnosis allows early appropriate treatment to be administered.

## Introduction

Reflex sympathetic dystrophy (RSD) is a complex regional pain syndrome characterized by variable dysfunctions of the musculoskeletal, skin and vascular systems [1]. Occasionally, the differential diagnosis includes psychiatric and functional disorders including malingering, frank psychosis and factitious illnesses in which the symptoms are self-induced.

We present a case of factitious lymphoedema (FL) mimicking RSD. This case report reiterates the need for a high level of suspicion when the signs and symptoms and, in this case, the treatment outcomes do not concur.

## Case presentation

**Figure 1:**
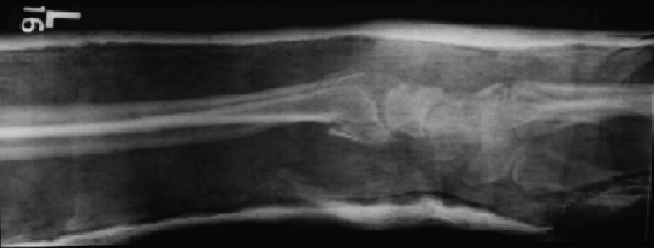
Reduced distal radius fracture in plaster cast.

A 33-year-old man sustained a left distal radius fracture, which was treated by manipulation and fixation in a plaster cast (Figure 1). He had fallen onto his outstretched arm while carrying out a domestic task. He had no other injuries and the fracture healed satisfactorily with minimal displacement and no neurovascular deficit.

**Figure 2:**
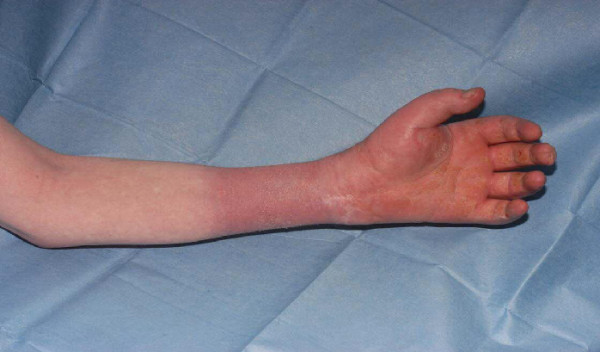
Skin changes on the ventral surface.

**Figure 3:**
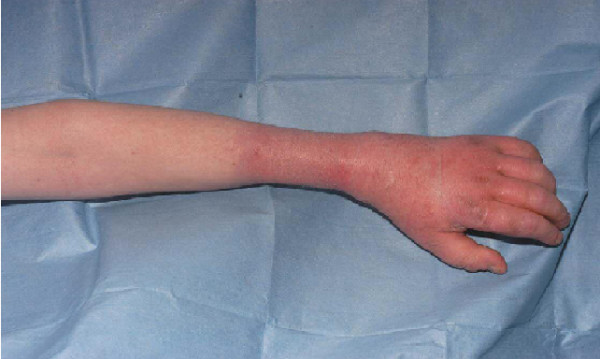
Skin changes on the dorsal surface.

Twenty-two months after discharge the patient presented to the accident and emergency department reporting a 2-day history of severe burning pain in his left forearm. His forearm was discoloured, swollen and very itchy (Figures 2 and 3). His hand was neurovascularly intact but he had a reduced range of movement and limitation of hand function due to stiffness and pain.

Physiotherapy including manipulative exercises and volar splints was tried initially, but the patient continued to suffer pain in the arm despite maximal opioid analgesia. He eventually required a Bier's sympathetic block, which relieved the pain, but his symptoms and signs recurred shortly after discharge.

It became apparent on subsequent attendances that the distribution of the swelling and discolouration of his forearm was more in keeping with the application of a ligature or proximal compress, resulting in subsequent swelling and discolouration. There was a clear line of demarcation on the mid-forearm and it was noted that the position of this line of demarcation varied in different consultations.

The patient had a history of excessive drinking and later, during psychiatric evaluation, he claimed to have been applying a ligature in order to relieve the pain in his wrist. He was referred for psychiatric treatment and made a full recovery.

## Discussion

This is a case of FL caused by intermittent application of a tourniquet to the forearm in a patient with an underlying psychiatric illness. The patient was thought to have RSD because of his presentation and his previous distal radius fracture.

RSD can occur after an injury to or operation on a limb. The incidence is estimated at 5% to 15% after all injuries [2]; the reported incidence of RSD in prospective studies of Colles fractures is 7% to 35% [3]. RSD can cause severe disability, with only one in five patients able to fully resume prior activities [2].

The signs of RSD include pain, oedema, stiffness and discolouration. There is usually an intense and burning pain, out of proportion to the injury and affecting the entire extremity. The pain may persist after the stimulus has been removed (hyperpathia), be present with light touch (allodynia) and be aggravated by movement. Oedema is usually one of the earliest findings, stiffness may occur and discolouration may vary from intense erythema to cyanosis or be pale, purple or grey. Treatment is supportive with physiotherapy, and pain control and the complete return of hand function are the goals. The approach to treatment depends largely on the specialty of the treating physician but options include sympathetic blocks, sympatholytic drugs and anti-inflammatory drugs. Calcitonin, which is available as a nasal spray, has been reported to reverse the inflammatory changes and reduce pain in early RSD, especially in patients with hyperdynamic blood flow [4].

FL can be caused by tourniquets, blows to the arm or repeated skin irritation, usually in patients with known psychiatric conditions [5,6]. FL results in symptoms and signs suggestive of RSD and a delay in diagnosis results in inappropriate treatment. The patient in this case did not have a known medical history of psychosis, behavioural disorder, self-harm or other psychiatric conditions that would have suggested that his presentation was FL not RSD. Clinical suspicion from observations of his arm during treatment and variation in the presentation led to a formal psychiatric evaluation that diagnosed FL.

## Conclusion

A high level of suspicion and early initiation of psychotherapy can result in effective treatment of this condition.

## Abbreviations

FL: factitious lymphoedema; RSD: reflex sympathetic dystrophy.

## Competing interests

The authors declare that they have no competing interests.

## Consent

Written informed consent was obtained from the patient for publication of this case report and any accompanying images. A copy of the written consent is available for review by the Editor-in-Chief of this journal.

## Authors' contributions

NN carried out case preparation, and wrote and edited the case report, HAPA identified the case for publication and carried out case preparation, DW was lead consultant and carried out case preparation.
